# Statin Use, Cholesterol Level, and Mortality Among Females With Breast Cancer

**DOI:** 10.1001/jamanetworkopen.2023.43861

**Published:** 2023-11-17

**Authors:** Mika O. Murto, Niklas Simolin, Otso Arponen, Aino Siltari, Miia Artama, Kala Visvanathan, Arja Jukkola, Teemu J. Murtola

**Affiliations:** 1 Department of General Surgery, Tays Cancer Centre, Tampere, Finland; 2 Faculty of Medicine and Health Technology, University of Tampere, Tampere, Finland; 3 Department of Radiology, Tampere University Hospital, Tampere, Finland; 4 Department of Pharmacology, Faculty of Medicine, University of Helsinki, Helsinki, Finland; 5 Department of Health Protection, Finnish Institute for Health and Welfare, Tampere, Finland; 6 Department of Epidemiology, Johns Hopkins Bloomberg School of Public Health, Baltimore, Maryland; 7 Department of Oncology, Johns Hopkins School of Medicine, Baltimore, Maryland; 8 Department of Oncology, Tampere University Hospital, Tampere University, Tays Cancer Centre, Tampere, Finland; 9 Department of Urology, Tays Cancer Centre, Tampere, Finland

## Abstract

**Importance:**

Several studies have reported an association between the use of statins and breast cancer (BC) mortality. However, most of these studies did not take into account the underlying cholesterol level.

**Objective:**

To investigate the association between serum cholesterol, statin use, and BC mortality.

**Design, Setting, and Participants:**

This cohort study included females with invasive BC that was newly diagnosed between January 1, 1995, and December 31, 2013, in Finland. The cohort had available hormone receptor data and at least 1 cholesterol measurement. All data were obtained from Finnish national registries. Statistical analyses were performed from January to May 2022.

**Exposure:**

Use of statins; statin dose; and serum cholesterol, low-density lipoprotein, high-density lipoprotein, and triglyceride levels measured separately before and after BC diagnosis.

**Main Outcomes and Measures:**

Breast cancer mortality and overall mortality between date of BC diagnosis and December 31, 2015.

**Results:**

A total of 13 378 female patients with BC (median [IQR] age, 62 [54-69] years) participated in the study. The median (IQR) follow-up was 4.5 (2.4-9.8) years after BC diagnosis, during which 16.4% of patients died and 7.0% died of BC. Prediagnostic statin use was a risk factor for BC death even after adjustment for total cholesterol level (hazard ratio [HR], 1.22; 95% CI, 1.02-1.46; *P* = .03). Reduced risk for BC death was seen for postdiagnostic statin use (HR, 0.85; 95% CI, 0.73-1.00; *P* = .05). The risk reduction was robust in participants whose cholesterol level decreased after starting statins (HR, 0.49; 95% CI, 0.32-0.75; *P* = .001) but was nonsignificant if cholesterol level did not subsequently decrease (HR, 0.69; 95% CI, 0.34-1.40; *P* = .30). Reduced BC mortality among statin users was also observed in females with estrogen receptor–positive tumors (HR, 0.82; 95% CI, 0.68-0.99; *P* = .03). Overall mortality was lower among statin users vs nonusers when adjusted for serum cholesterol level (HR, 0.80; 95% CI, 0.72-0.88; *P* < .001).

**Conclusions and Relevance:**

Results of this cohort study showed that postdiagnostic use of statins was associated with reduced BC mortality compared with nonuse, and the risk was associated with subsequent change in serum cholesterol level. This finding suggests that cholesterol-lowering interventions with statins may be beneficial for patients with BC.

## Key Points

**Question:**

Does the use of statins affect breast cancer (BC) mortality?

**Findings:**

In this cohort study of 13 378 females diagnosed with BC, postdiagnostic statin use was associated with a lower risk of BC death only if the median total cholesterol level decreased subsequently.

**Meaning:**

Findings of this study suggest that cholesterol-lowering interventions with statins may be beneficial for patients with BC.

## Teaser

This cohort study evaluates the association of serum cholesterol level and statin use with breast cancer (BC)–specific and overall mortality in females with invasive BC.

## Introduction

Over 2 million people develop breast cancer (BC) and over 680 000 people die of BC every year, making BC the most common cancer and cause of cancer death among females worldwide.^1^ In Finland, approximately 5000 new cases of BC are diagnosed and 900 females die of the disease yearly.^2^ Although survival rates for localized BC are high, many cases still recur,^3^ and survival rates for metastatic disease are poor.

3-Hydroxy-3-methylglutaryl coenzyme A (HMG-CoA) reductase is a key enzyme in the mevalonate pathway, which produces cholesterol and nonsteroidal isoprenoid compounds.^4^ The HMG-CoA reductase inhibitors, commonly known as statins, are well-tolerated drugs and widely used in primary and secondary prevention of cardiovascular disease (CVD). They are the first-line medication for atherosclerotic CVD and hyperlipidemia as well as secondary prevention for patients developing CVD. Several studies have shown that statins may be beneficial for BC survival.^5,6,7,8,9^ This beneficial risk association has not been attributed to any specific BC type or treatment. The inhibition of HMG-CoA reductase may affect cancer indirectly by lowering serum lipoproteins^10^ or directly through intracellular signaling pathways. In vitro statins cause cell cycle arrest by blocking DNA replication phase.^11^ In BC cells, statins have anti-invasive and antiangiogenic effects^12^ and induce apoptosis through HMG-CoA reductase inhibition.^13^ Previous studies, including a Finnish population-based cohort study, suggest that statins are associated with improved BC survival^5,6,7^; however, a large systematic review found that the pooled association was not robust and was heterogeneous.^8^ It has been suggested that statins may be beneficial for BC survival in patients with metastatic disease.^9^

Moreover, serum cholesterol level may have a role in BC outcome. Oxysterol 27-hydroxycholesterol, the main cholesterol metabolite, promotes tumor growth and metastases in BC mouse models^14^ and has been associated with BC mortality in females with low estrogen levels.^15^ Cholesterol is also a required precursor for estrogen biosynthesis; high estrogen levels are associated with increased risk of BC.^16^ However, previous studies did not take into account simultaneous cholesterol levels or changes due to statin use, contributing to the heterogeneity of research results, and the independent roles of statin use and cholesterol levels in BC outcomes remain unknown. In this study, we aimed to investigate the association between serum cholesterol, statin use, and BC mortality in a nationwide Finnish cohort.

## Methods

### Study Cohort

In accordance with Finnish national laws and regulations, this retrospective, population-based cohort study was exempt from ethics committee review and the requirement for informed consent because it was carried out using routinely collected registry data from national registries. Each registry approved the study protocol. We collected and combined the data from national governmental registries with permission from the administrator of each registry to use personal identification numbers. We followed the Strengthening the Reporting of Observational Studies in Epidemiology (STROBE) reporting guideline.

The cohort consisted of all females who were newly diagnosed with invasive BC between January 1, 1995, and December 31, 2013, in Finland. We identified BC cases from the Finnish Cancer Registry, which contains cancer diagnoses from all pathology laboratories and health care units in the country, covering 99% of cancer cases nationally.^17,18^ These BC cases had *International Statistical Classification of Diseases and Related Health Problems, Tenth Revision (ICD-10)* diagnosis codes of C50.1 to C50.9. Available data in the registry were patient age; diagnosis date; tumor extent (available for 85.6% of cases) categorized as localized, locally advanced, and metastatic; primary BC treatment (available for 99.6% of cases); and tumor histological characteristics (available for 99.9% of cases). Tumor grade was not available.

We limited the cohort to cases with available hormone receptor data and at least 1 cholesterol measurement, resulting in a well-characterized cohort of females with invasive BC. Compared with included females, excluded females had higher BC-specific and overall mortality, a greater proportion with metastatic or unknown tumor extent at diagnosis, and a slightly lower proportion with curative-intent surgery as primary treatment (eTable 1 in Supplement 1).

We retrieved the dates and causes of deaths from Statistics Finland, which registers information from death certificates with almost 99% accuracy in BC cases,^19^ until December 31, 2015. We considered a death to be caused by BC if any of the BC-specific *ICD-10* diagnosis codes (C50.1-C50.9) was listed as the primary cause. All deaths, regardless of the cause, were included in the analyses of overall survival.

In Finland, nationwide mammography screening is provided every 2 years to all females between 50 and 69 years of age. National attendance to these mammograms is approximately 82%.^17,19^ The mammography results are registered in the Mass Inspection Registry, from which we obtained the number of mammography screenings before BC diagnosis.

### Serum Cholesterol Level and Hormone Receptor Status

We gathered serum cholesterol and hormone receptor data from the laboratory databases of Pirkanmaa, Southwest Finland, and Helsinki-Uusimaa Hospital Districts. The databases included the dates and results of each lipoprotein measurement from 1995 to 2015. The lipid parameter data included total cholesterol, high-density lipoprotein (HDL), low-density lipoprotein (LDL), and triglyceride concentrations. We identified median cholesterol concentrations for each calendar year separately using all measurements during that year. We used the latest available median for the years with no available measurements. We categorized cholesterol and other lipid parameter levels as normal or elevated according to the recommended cardiovascular target levels: 193.05 mg/dL for total cholesterol, 46.33 mg/dL for HDL, 115.83 mg/dL for LDL, and 150.44 mg/dL for triglycerides. To convert total cholesterol, HDL, and LDL to millimoles per liter, multiply by 0.0259; triglycerides to millimoles per liter, multiply by 0.0113.

We estimated the changes in blood cholesterol levels after the start of statin use in the postdiagnostic period by calculating the median cholesterol levels before and after the first year of statin purchase. Cholesterol measurements from the first year of statin use were excluded because the exact time for the initiation of drug use was not known based on purchase data. Participants were stratified by whether the median total cholesterol level had decreased or stayed the same or increased after the first year of statin purchase.

Data on hormone receptor, lipoprotein, and triglyceride measurements included estrogen receptor (ER), progesterone receptor (PR), and *ERBB2* (formerly *HER2*) status, which has been in clinical use in Finland since 2002. We deemed ER and PR as negative if less than 10% of tumor cells were stained and as positive if 10% or more of tumor cells were stained.^20,21^ We ascertained *ERBB2* status from pathology reports.

### Comorbidities

The Care Register for Health Care includes all diagnoses and procedures from inpatient and outpatient hospital visits. Data on hypertension, hypercholesterolemia, coronary artery disease, and diabetes from 1995 to 2013 were collected using *ICD-10* codes (eTable 2 in Supplement 1). Primary health care visits are not included in the database. We calculated the Charlson Comorbidity Index (CCI)^22^ based on diagnoses in the Care Register for Health Care database and the drug purchase data from the Social Insurance Institution database.

### Statin Use

The Social Insurance Institution maintains a national drug prescription database of physician-prescribed drug purchases reimbursed as part of the national health insurance. This prescription database does not record over-the-counter purchases or drugs dispensed during hospitalizations. Rather, it records information on each drug purchase, including the purchase date, Anatomical Therapeutic Chemical classification of the drug (eTable 3 in Supplement 1), pill strength, and package size. We linked the study cohort to the Social Insurance Institution database to collect person-level information on cholesterol-lowering, antihypertensive, antidiabetic, and anticoagulant medication purchases between 1995 and 2015.

We added together all statin purchases in a given year for a yearly total milligram amount of use for each statin, and we standardized the cumulative use between different statins by dividing the yearly purchased amount (in milligrams) with the drug-specific defined daily dose (DDD) as listed by the World Health Organization.^23^ All cohort participants were categorized as statin nonusers until the year of the first record of statin purchase. We considered each year with recorded statin purchases to be a year of use regardless of the purchased amount. To limit bias due to selective discontinuation of statin use in the palliative phase of cancer care, we did not allow participant status to revert to nonuser status after the first statin purchase. To estimate the yearly dose of statin use, we calculated an intensity variable by dividing the cumulative yearly DDD amount with the cumulative duration of use.

### Statistical Analysis

The primary analysis was the comparison of risk of BC death between statin users and nonusers and by total cholesterol level. Secondary analyses were performed for LDL, HDL, and triglyceride levels adjusted for statin use or cholesterol level and stratified by statin dose, subsequent cholesterol decrease, and hormone receptor status. All subgroup analyses were determined a priori. Overall survival was analyzed as a secondary outcome.

We used the Cox proportional hazards regression model to evaluate the risk of BC death and overall death (hazard ratio [HR]) while adjusting for patient age; tumor histological characteristics; tumor extent at diagnosis; primary treatment (curative-intent surgery vs other oncological treatments); number of mammography screenings; CCI; and any use of antidiabetic, anticoagulant, antihypertensive, or antihormonal therapy. A detailed description of all exposure and outcome variables used in the analyses is provided in the eMethods in Supplement 1. Follow-up started on the date of BC diagnosis and continued until death, emigration, or common closing date of December 31, 2015, whichever occurred first. Tumor histological features and tumor extent were included, as they are established clinical characteristics; primary treatment was included because it reflects the participants’ general health and tumor extent and hence estimated outcome; mammography screening was included because cancer screening provides lead time in cancer detection that affects survival times; and CCI and use of other drugs were included because they reflect the participants’ general health, likelihood to start statin use, and overall estimated outcome. In a sensitivity analysis, we further adjusted the analysis for total cholesterol level or statin use to estimate the role of cholesterol while adjusting for simultaneous statin use and vice versa. Exploratory subgroup analyses were stratified by tumor extent and ER, PR, and *ERBB2* statuses.

We analyzed statin use by timing the use in relation to BC diagnosis. Prediagnostic statin exposure time included all purchases from 1995 until the year of BC diagnosis. Postdiagnostic statin use included all purchases starting from the year of BC diagnosis until the end of the follow-up period in 2015.

To minimize immortal time bias,^24^ we analyzed postdiagnosis statin use and serum cholesterol level as time-dependent variables; for each follow-up year starting from the year of BC diagnosis, statin use and cholesterol levels were updated on a yearly basis. All participants accumulated follow-up time as nonusers until the year of the first statin purchase. We used lag-time analyses to control for protopathic bias and to estimate possible delayed outcomes of statin use within 1 to 5 years after use.^25^ To control for the bias associated with selective discontinuation of statin use at the terminal phase of cancer, we did not allow statin use status to return to nonuser status after the first purchase.^25^ We also formed similar time-dependent variables for the use of antidiabetic, anticoagulant, and antihypertensive drugs as well as for yearly median cholesterol levels.

We regarded *P* < .05 as statistically significant. All statistical tests were 2-sided. Statistical analyses were performed from January to May 2022 with IBM SPSS for Windows, version 26.0 (IBM Corp).

## Results

The cohort consisted of 13 378 females with BC, with a median (IQR) age of 62 (54-69) years. The median (IQR) follow-up was 4.5 (2.4-9.8) years after BC diagnosis, during which 16.4% of patients died and 7.0% of patients died of BC. A total of 31.2% of patients had an elevated median total cholesterol level (>193.05 mg/dL) before the BC diagnosis, and 50.3% of patients had elevated total cholesterol levels after the diagnosis. Among all patients, 40.7% had ever used statins (Table 1).

**Table 1.** Characteristics of 13 378 Females With a Breast Cancer (BC) Diagnosis in Finland From 1995 to 2013

Characteristic	Cholesterol level before BC diagnosis, No. (%)		Statin drug use before BC diagnosis, No. (%)		Cholesterol level after BC diagnosis, No. (%)		Statin drug use after BC diagnosis, No. (%)	
	Normal	Elevated^a^	No	Yes	Normal	Elevated^a^	No	Yes
Total No. of participants	3199 (23.9)	4170 (31.2)	10 177 (76.1)	3201 (23.9)	6291 (47.0)	7087 (53.0)	7927 (59.3)	5451 (40.7)
Median follow-up time (IQR), y	3.32 (1.29-4.80)	3.13 (1.42-5.39)	5.13 (2.47-8.80)	3.05 (1.38-5.38)	4.34 (2.05-7.88)	4.63 (2.13-8.13)	4.38 (2.13-7.80)	4.63 (2.13-8.22)
Deaths								
BC deaths	212 (6.6)	270 (6.5)	710 (7.0)	225 (7.0)	481 (7.6)	454 (6.4)	601 (7.6)	334 (6.1)
BC mortality per 1000 person-years	19.95	17.53	11.34	18.84	14.00	11.30	13.92	10.66
Overall deaths	593 (18.5)	630 (15.1)	1573 (15.5)	621 (19.4)	1267 (20.1)	927 (13.1)	1291 (16.3)	903 (16.6)
Overall mortality per 1000 person-years	55.80	40.91	25.13	51.99	36.89	23.07	29.90	28.81
Age at BC diagnosis								
Median age (IQR), y	66 (57-76)	66 (58-73)	60 (52-68)	69 (63-77)	64 (55-74)	62 (54-68)	60 (52-68)	66 (58-74)
≤39	13 (0.4)	6 (0.1)	53 (0.5)	1 (0)	34 (0.5)	20 (0.3)	52 (0.7)	2 (0)
40-55	396 (12.4)	285 (6.8)	1314 (12.9)	65 (2.0)	713 (11.3)	666 (9.4)	1218 (15.4)	161 (3.0)
≥56	2790 (87.2)	3879 (93.0)	8810 (86.6)	3135 (97.9)	5544 (88.1)	6401 (90.3)	6657 (84.0)	5288 (97.0)
Tumor extent at diagnosis								
Localized	1726 (54.0)	2265 (54.3)	5539 (54.4)	1733 (54.1)	3443 (54.7)	3829 (54.0)	4309 (54.4)	2963 (54.4)
Locally advanced	1092 (34.1)	1459 (35.0)	3637 (35.7)	1078 (33.7)	2125 (33.8)	2590 (36.5)	2808 (35.4)	1907 (35.0)
Metastatic	232 (7.3)	253 (6.1)	560 (5.5)	227 (7.1)	409 (6.5)	378 (5.3)	468 (5.9)	319 (5.9)
Unknown	149 (4.7)	193 (4.6)	441 (4.3)	163 (5.1)	314 (5.0)	290 (4.1)	342 (4.3)	262 (4.8)
Tumor histological characteristics								
Invasive ductal	2354 (73.6)	3086 (74.0)	7502 (73.7)	2366 (73.9)	4630 (73.6)	5238 (73.9)	5864 (74.0)	4004 (73.5)
Invasive lobular	597 (18.7)	762 (18.3)	1974 (19.4)	589 (18.4)	1185 (18.8)	1378 (19.4)	1500 (18.9)	1063 (19.5)
Other	246 (7.7)	322 (7.7)	700 (6.9)	245 (7.7)	474 (7.5)	471 (6.6)	562 (7.1)	383 (7.0)
Unknown	2 (0.1)	0	0	1 (0)	2 (0)	0	1 (0)	1 (0)
Hormone receptor status								
ER positive	2748 (85.9)	3623 (86.9)	8443 (83.0)	2736 (85.5)	5235 (83.2)	5944 (83.9)	6606 (83.3)	4573 (83.9)
ER negative	386 (12.1)	501 (12.0)	1270 (12.5)	371 (11.6)	763 (12.1)	878 (12.4)	1000 (12.6)	641 (11.8)
ER status unknown	65 (2.0)	46 (1.1)	464 (4.6)	94 (2.9)	293 (4.7)	265 (3.7)	321 (4.0)	237 (4.3)
PR positive	2248 (70.3)	2892 (69.4)	6695 (65.8)	2201 (68.8)	4252 (67.6)	4644 (65.5)	5259 (66.3)	3637 (66.7)
PR negative	872 (27.3)	1205 (28.9)	2952 (29.0)	878 (27.4)	1707 (27.1)	2123 (30.0)	2309 (29.1)	1521 (27.9)
PR status unknown	79 (2.5)	73 (1.8)	530 (5.2)	122 (3.8)	332 (5.3)	320 (4.5)	359 (4.5)	293 (5.4)
*ERBB2* positive^b^	934 (29.2)	1118 (26.8)	2827 (27.8)	920 (28.7)	1712 (27.2)	2035 (28.7)	2230 (28.1)	1517 (27.8)
*ERBB2* negative^b^	2171 (67.9)	2962 (71.0)	6923 (68.0)	2147 (67.1)	4312 (68.5)	4758 (67.1)	5374 (67.8)	3696 (67.8)
*ERBB2* status unknown^b^	94 (2.9)	90 (2.2)	427 (4.2)	134 (4.2)	267 (4.2)	294 (4.1)	323 (4.1)	238 (4.4)
Triple negative: ER negative, PR negative, *ERBB2* negative	211 (6.6)	287 (6.9)	671 (6.6)	198 (6.2)	424 (6.7)	445 (6.3)	535 (6.7)	334 (6.1)
Mammography screening history								
Any	1969 (61.6)	2935 (70.4)	6952 (68.3)	2077 (64.9)	3874 (61.6)	5155 (72.7)	5277 (66.6)	3752 (68.8)
Primary treatment								
Curative-intent surgery	2357 (73.7)	3187 (76.4)	7933 (78.0)	2331 (72.8)	4742 (75.4)	5522 (77.9)	6129 (77.3)	4135 (75.9)
Other or unknown	842 (26.3)	983 (23.6)	2244 (22.0)	870 (27.2)	1549 (24.6)	1565 (22.1)	1798 (22.7)	1316 (24.1)
Antihormonal therapy								
Any	1087 (34.0)	1351 (32.4)	4219 (41.5)	952 (29.7)	2439 (38.8)	2732 (38.5)	3382 (42.7)	1789 (32.8)
Comorbidities								
CCI, median (IQR)	0 (0-1)	0	0	0 (0-1)	0 (0-1)	0	0	0 (0-1)
CCI, mean (IQR)	0.74 (0-1)	0.60 (0-0)	0.66 (0-0)	0.70 (0-1)	0.76 (0-1)	0.59 (0-0)	0.64 (0-0)	0.71 (0-1)
Hypertension	2470 (77.2)	3111 (74.6)	6772 (66.5)	2830 (88.4)	4855 (77.2)	4747 (67.0)	4895 (61.8)	4707 (86.4)
Diabetes	811 (25.4)	682 (16.4)	1345 (13.2)	1030 (32.2)	1608 (25.6)	767 (10.8)	735 (9.3)	1640 (30.1)
CVD	275 (8.6)	165 (4.0)	232 (2.3)	383 (12.0)	436 (6.9)	179 (2.5)	107 (1.3)	508 (9.3)

Abbreviations: CCI, Charlson Comorbidity Index; CVD, cardiovascular disease; ER, estrogen receptor; PR, progesterone receptor.

^a^ Elevated level of total cholesterol was higher than 193.05 mg/dL (to convert to millimoles per liter, multiply by 0.0259).

^b^ *ERBB2* status was available from 2002 onward.

None of the 4 lipid parameters before or after the BC diagnosis were associated with BC death in multivariable-adjusted analysis (Table 2; eTable 4 in Supplement 1). Further adjustment for simultaneous use of statins did not change the result.

**Table 2.** Risk of Breast Cancer (BC) Death by Serum Total Cholesterol and Low-Density Lipoprotein (LDL) Levels Before and After BC Diagnosis

Lipid profile status	Total cholesterol level				LDL level			
	No. of BC deaths/total No. of participants (%)	HR for BC death (95% CI)			No. of BC deaths/total No. of participants (%)	HR for BC death (95% CI)		
		Age-adjusted	Multivariable-adjusted^a^	Multivariable + statin use–adjusted		Age-adjusted	Multivariable-adjusted^a^	Multivariable + statin use–adjusted
**Lipid profile status before BC diagnosis** ^b^								
Under reference value^c^	212/3199 (6.6)	1 [Reference]	1 [Reference]	1 [Reference]	158/2619 (6.0)	1 [Reference]	1 [Reference]	1 [Reference]
Over reference value^c^	270/4170 (6.5)	0.90 (0.75-1.07)	0.99 (0.82-1.20)	1.01 (0.83-1.22)	227/3279 (6.9)	0.90 (0.74-1.10)	0.97 (0.78-1.20)	0.99 (0.80-1.23)
First tertile^d^	163/2485 (6.6)	1 [Reference]	1 [Reference]	1 [Reference]	145/2093 (6.9)	1 [Reference]	1 [Reference]	1 [Reference]
Second tertile^d^	156/2448 (6.4)	0.96 (0.77-1.20)	1.12 (0.89-1.41)	1.15 (0.91-1.44)	122/1842 (6.6)	1.01 (0.80-1.29)	1.09 (0.85-1.41)	1.13 (0.87-1.45)
Third tertile^d^	163/2436 (6.7)	0.94 (0.76-1.17)	1.05 (0.83-1.32)	1.06 (0.85-1.34)	118/1963 (6.0)	0.89 (0.69-1.13)	0.94 (0.73-1.22)	0.97 (0.75-1.25)
**Lipid profile status after BC diagnosis**								
Under reference value^c^	481/6291 (7.6)	1 [Reference]	1 [Reference]	1 [Reference]	464/5631 (8.2)	1 [Reference]	1 [Reference]	1 [Reference]
Over reference value^c^	454/7087 (6.4)	0.92 (0.81-1.06)	1.07 (0.92-1.24)	1.05 (0.91-1.22)	285/4860 (5.9)	0.81 (0.70-0.95)	0.86 (0.73-1.02)	0.85 (0.72-1.01)
First tertile^e^	394/4648 (8.5)	1 [Reference]	1 [Reference]	1 [Reference]	332/3708 (9.0)	1 [Reference]	1 [Reference]	1 [Reference]
Second tertile^e^	250/4351 (5.7)	0.77 (0.66-0.91)	0.85 (0.71-1.01)	0.83 (0.69-0.99)	218/3283 (6.6)	0.89 (0.74-1.06)	1.01 (0.83-1.22)	0.99 (0.81-1.20)
Third tertile^e^	291/4378 (6.6)	0.85 (0.72-0.99)	1.02 (0.86-1.22)	1.00 (0.84-1.20)	199/3495 (5.7)	0.76 (0.63-0.92)	0.85 (0.69-1.05)	0.84 (0.69-1.04)

Abbreviations: HDL, high-density lipoprotein; HR, hazard ratio.

SI conversion factor: To convert HDL, LDL, and total cholesterol to millimoles per liter, multiply by 0.0259; triglycerides to millimoles per liter, multiply by 0.0113.

^a^ Calculated using a Cox proportional hazards regression model adjusted for age at BC diagnosis, number of mammography screening rounds attended before BC diagnosis, tumor extent, tumor histological characteristics, primary treatment, coronary artery disease, diabetes, hypertension, Charlson Comorbidity Index, hormone receptor status, and use of antihormonal therapy after BC diagnosis.

^b^ Analysis limited to participants with at least 1 cholesterol or lipid measurement available between 1995 and the year of BC diagnosis.

^c^ Cut-off values: total cholesterol higher than 193.05; triglycerides higher than 150.44; HDL lower than 46.33; and LDL higher than 115.83 mg/dL.

^d^ Total cholesterol tertiles before BC diagnosis: first, 185.33 mg/dL or less; second, higher than 185.33 to 214.29 mg/dL; and third, higher than 214.29 mg/dL. LDL tertiles before BC diagnosis: first, 100.39 mg/dL or less; second, higher than 100.39 to 125.87 mg/dL; and third, higher than 125.87 mg/dL.

^e^ Total cholesterol tertiles after BC diagnosis: first, 181.47 mg/dL or less; second, higher than 181.47 to 212.36 mg/dL; and third, higher than 212.36 mg/dL. Triglyceride tertiles after BC diagnosis: first, 84.07 mg/dL or less; second, higher than 84.07 to 121.68 mg/dL; and third, higher than 121.68 mg/dL. HDL tertiles after BC diagnosis: first, 56.37 mg/dL or less; second, higher than 56.37 to 71.43 mg/dL; and third, higher than 71.43 mg/dL. LDL tertiles after BC diagnosis: first, 100.39 mg/dL or less; second, higher than 100.39 to 128.69 mg/dL; and third, higher than 128.69 mg/dL.

In multivariable-adjusted analysis, statin use before BC diagnosis was associated with an increased risk of BC death compared with nonuse (HR, 1.41; 95% CI, 1.18-1.68; *P* < .001) (Table 3). Further adjustment for prediagnostic lipid parameters attenuated the increased risk, albeit not entirely. The risk increase by prediagnostic statin use was not dose dependent. Prediagnostic statin use was a risk factor for BC death even after adjustment for total cholesterol level (HR, 1.22; 95% CI, 1.02-1.46; *P* = .03) (Table 3).

**Table 3.** Risk of Breast Cancer (BC) Death by Statin Use and With Adjustments Before and After BC Diagnosis

Statin use status	No. of BC deaths/total No. of participants (%)	HR for BC death (95% CI)					
		Age-adjusted	Multivariable-adjusted^a^	Additional lipid parameter adjustments^b^			
				Total cholesterol	LDL	HDL	Triglycerides
**Use before BC diagnosis**							
None	710/10 177 (7.0)	1 [Reference]	1 [Reference]	1 [Reference]	1 [Reference]	1 [Reference]	1 [Reference]
Any	225/3201 (8.0)	1.21 (1.03-1.42)	1.41 (1.18-1.68)	1.22 (1.02-1.46)	1.24 (1.04-1.49)	1.21 (1.02-1.45)	1.23 (1.03-1.47)
Intensity of statin use, DDD/y							
First tertile^c^	73/1057 (6.9)	1.03 (0.80-1.31)	1.15 (0.89-1.49)	1.01 (0.77-1.31)	1.02 (0.79-1.33)	1.00 (0.77-1.30)	1.02 (0.79-1.33)
Second tertile^c^	82/1056 (7.8)	1.29 (1.02-1.63)	1.60 (1.24-2.05)	1.37 (1.07-1.77)	1.40 (1.09-1.81)	1.36 (1.06-1.75)	1.38 (1.07-1.78)
Third tertile^c^	65/1057 (6.1)	1.25 (0.96-1.62)	1.47 (1.10-1.97)	1.27 (0.95-1.70)	1.29 (0.96-1.73)	1.26 (0.94-1.68)	1.27 (0.95-1.70)
**Use after BC diagnosis**							
None	601/7927 (7.6)	1 [Reference]	1 [Reference]	1 [Reference]	1 [Reference]	1 [Reference]	1 [Reference]
Any	334/5451 (6.1)	1.04 (0.91-1.20)	0.95 (0.80-1.11)	0.85 (0.73-1.00)	0.84 (0.72-0.99)	0.85 (0.72-0.99)	0.85 (0.72-1.00)
Intensity of statin use, DDD/y							
First tertile^d^	124/1628 (7.6)	1.21 (0.99-1.47)	1.14 (0.92-1.42)	1.03 (0.83-1.28)	1.04 (0.83-1.29)	1.03 (0.83-1.28)	1.03 (0.83-1.28)
Second tertile^d^	99/1585 (6.2)	0.96 (0.77-1.20)	0.82 (0.64-1.05)	0.76 (0.59-0.97)	0.73 (0.57-0.94)	0.75 (0.59-0.96)	0.75 (0.59-0.97)
Third tertile^d^	60/1603 (3.7)	0.84 (0.66-1.08)	0.76 (0.58-1.01)	0.68 (0.51-0.90)	0.66 (0.50-0.87)	0.68 (0.51-0.89)	0.68 (0.51-0.89)

Abbreviations: DDD, defined daily dose; HDL, high-density lipoprotein; HR, hazard ratio; LDL, low-density lipoprotein.

^a^ Calculated using a Cox proportional hazards regression model adjusted for age at BC diagnosis, number of mammography screening rounds attended before BC diagnosis, tumor extent, tumor histological characteristics, primary treatment, coronary artery disease, diabetes, hypertension, Charlson Comorbidity Index, hormone receptor status, and use of antihormonal therapy after BC diagnosis.

^b^ Cut-off values: total cholesterol higher than 193.05; triglycerides higher than 150.44; HDL lower than 46.33; and LDL higher than 115.83 mg/dL (to convert total cholesterol, HDL, and LDL to millimoles per liter, multiply by 0.0259; triglycerides to millimoles per liter, multiply by 0.0113).

^c^ Statin tertiles before BC diagnosis: first, higher than 0 to 111.6667 DDD/y; second, higher than 111.6667 to 192.8148 DDD/y; and third, higher than 192.8148 DDD/y.

^d^ Statin tertiles after BC diagnosis: first, higher than 0 to 130.6667 DDD/y; second, higher than 130.6667 to 235.2000 DDD/y; and third, higher than 235.2000 DDD/y.

Conversely, postdiagnostic statin use was not associated with BC mortality before adjustment for lipid parameters. After this adjustment, there was a statistically significant inverse association with BC death regardless of the lipid parameter for which we adjusted (eg, for total cholesterol: HR, 0.85 [95% CI, 0.73-1.00]; *P* = .05 and for LDL: HR, 0.84; 95% CI, 0.72-0.99). The risk reduction by postdiagnostic statin use became greater along with increasing intensity of use (eg, for LDL: HR, from 0.73 [95% CI, 0.57-0.94] to 0.66 [95% CI, 0.50-0.87]) (Table 3).

In total, 980 patients began statin use after BC diagnosis. Median cholesterol level decreased subsequently in 781 participants, whereas the level remained similar or increased compared with prestatin purchase levels in 199 participants. When stratified by change in cholesterol level after the initiation of statin use, postdiagnostic statin use was associated with a lowered risk of BC death when the median total cholesterol decreased subsequently (HR, 0.49; 95% CI, 0.32-0.75; *P* = .001). The risk decrease was nonsignificant if cholesterol levels did not decrease or increased after the start of statin use (HR, 0.69; 95% CI, 0.34-1.40; *P* = .30) (Table 4).

**Table 4.** Risk of Breast Cancer (BC) Death by Postdiagnostic Statin Use

Statin use status	No. of BC deaths/total No. of participants (%)	BC death			
		Age-adjusted		Multivariable-adjusted	
		HR (95% CI)	*P* value	HR (95% CI)	*P* value
Nonuser	604/7948 (7.6)	1 [Reference]	NA	1 [Reference]	NA
Postdiagnostic statin user; cholesterol level decreased	24/781 (3.1)	0.57 (0.38-0.84)	.005	0.49 (0.32-0.75)	.001
Postdiagnostic statin user; cholesterol level not decreased	7/199 (3.5)	0.90 (0.48-1.68)	.73	0.69 (0.34-1.40)	.30

Abbreviations: HR, hazard ratio; NA, not applicable.

There was no association between BC death and postdiagnostic statin use in lag-time analyses with a 1-year, 3-year, or 5-year time lag (eTable 5 in Supplement 1). Elevated median cholesterol either before (HR, 0.80; 95% CI, 0.71-0.90; *P* < .001) or after (HR, 0.88; 95% CI, 0.80-0.97; *P* = .03) BC diagnosis was associated with a decreased risk of overall death, which did not change significantly after adjustment for statin use (HR, 0.81; 95% CI, 0.72-0.91; *P* = .02 and HR, 0.86; 95% CI, 0.78-0.94; *P* = .03, respectively) (eTables 6 and 7 in Supplement 1).

Statin use before BC diagnosis was associated with a higher risk of overall death (HR, 1.28; 95% CI, 1.15-1.43; *P* < .001) compared with risk for nonusers, and the risk increase was dose dependent. Statin users after BC diagnosis had a decreased risk of overall death (HR, 0.89; 95% CI, 0.80-0.98; *P* = .03). The risk decreased along with increasing intensity of statin use after adjustment for lipid parameters (serum cholesterol level: HR, 0.80; 95% CI, 0.72-0.88; *P* < .001) (eTable 8 in Supplement 1).

### Subgroup Analyses

Hormone receptor status or participation in mammography screening did not modify the association between statin use and BC mortality. The risk for BC death was approximately 20% lower among statin users compared with nonusers in each subgroup after adjusting for cholesterol level. However, the risk difference was statistically significant only in the ER-positive cases (HR, 0.82; 95% CI, 0.68-0.99; *P* = .03). In females with triple-negative BC (ER negative, PR negative, and *ERBB2* negative), no risk difference was observed between statin users and nonusers (eTable 9 in Supplement 1). These analyses were limited by small numbers; thus, they were only hypothesis generating.

Tumor extent modified the risk association. Lowered risk among statin users was observed in females with localized tumor extent at diagnosis (HR, 0.59; 95% CI, 0.41-0.86; *P* < .001). Among females with metastatic disease, statin use was associated with elevated mortality compared with nonuse (HR, 1.38; 95% CI, 1.01-1.87; *P* = .045) (eTable 9 in Supplement 1).

## Discussion

In this comprehensive cohort study, the inverse association between statin use and BC mortality was partly mediated by underlying cholesterol level. The risk decrease by statin use was clear only when the serum cholesterol level decreased simultaneously, although the subgroup analyses were limited by low statistical power. This finding suggests that serum cholesterol may be an important factor in BC and that the previously reported^26^ inverse association between statin use and BC mortality is likely mediated by underlying cholesterol level changes. Nevertheless, statin use after BC diagnosis was associated with a decreased risk for BC death even after adjustment for cholesterol level. The risk decreased further with increasing intensity of use, suggesting that statin use may affect estimated BC outcomes by mechanisms other than cholesterol reduction. The association did not persist in lag-time analyses, which suggests that the risk association was either short term or affected by protopathic bias. However, the lag-time analyses had lower statistical power than the main analysis.

Elevated serum cholesterol level was not associated with the risk of BC death after adjusting for statin use, which is in concordance with results of previous studies on the topic.^27,28^ To our knowledge, no published studies have explored the association between serum cholesterol and BC mortality. The well-established phenomenon of reverse causality (ie, advanced cancer resulting in spontaneous cholesterol decrease for years before cancer diagnosis or death^29,30^) may not be as evident for BC and with modern mammography screening practices, which have been factors in tumors being diagnosed at an earlier stage than before.

The beneficial role of cholesterol reduction is plausible given that in epidemiological studies, dietary cholesterol and hypercholesterolemia have been associated with increased risk of BC.^31,32^ Cholesterol synthesis is vital for cell proliferation. It is involved in the assembly and maintenance of cell membranes, modulating their function and fluidity. The primary cholesterol metabolite activates the ER, subsequently increasing BC cellular proliferation and tumor growth.^33^ Cholesterol is also a precursor for biosynthesis of estrogen, which in high levels is considered to be a mammary gland carcinogen.^14^ Oxysterol derivatives of cholesterol, such as 27-hydroxycholesterol, act as endogenous ER modulators and have been associated with worse outcomes in females with low estrogen.^15^

Statin use has been previously associated with reduced BC mortality, although research results were not conclusive.^5,8,33^ These studies did not take into account cholesterol change during statin use, which may be the reason for the discrepancies.

Inverse mortality association with statin use was observed in hormone receptor–positive cases but not in triple-negative cases. This finding suggests that hormone receptor positivity may have a role in the association between statins and BC. Tumor extent had a role, as the risk decrease was evident only in localized disease. In females with metastatic disease, the risk was conversely increased among statin users. Thus, statins may be beneficial only in females with early-stage BC. On the other hand, patients with metastatic disease have worse outcomes overall. It is plausible that interventions such as statins are unlikely to change the course of disease at this stage.

Unlike postdiagnostic use, prediagnostic statin use was associated with elevated risk for both BC death and overall death in a non–dose-dependent manner. This finding suggests that prediagnostic use may not be a beneficial factor in BC but may instead reflect worse overall health, which is plausible given that statins are commonly used in the secondary prevention of CVDs.

### Strengths and Limitations

Strengths of this study are the comprehensive national coverage and the well-defined cohort with detailed data on statin exposure. Serum cholesterol levels were available for analysis, providing us with better insight into the association between cholesterol level and statin use and allowing us to evaluate the outcome of statin-induced cholesterol change.

The study is limited by the lack of information on lifestyle factors, such as smoking, obesity, and physical activity, that are known to be associated with the risk of BC mortality.^34,35,36^ Furthermore, there was no information on socioeconomic status, which may be inversely associated with BC outcome^37^ and confound the observed risk associations. Additionally, there was no information on hormone replacement therapy in the prescription registry. Statin use was not randomized and therefore the possibility for residual bias remained despite adjustment for multiple potential confounding factors, such as clinical tumor characteristics, mammography screening, and comorbidity. The data on statin use were based on drug purchases, and we did not have information on whether the drugs were consumed. The interpretation of hormone receptor positivity from pathological diagnostic statements was sometimes ambiguous, especially for *ERBB2* status. This ambiguity may explain why the proportion of *ERBB2*-enriched tumors appeared high. These limitations and uncertainties affect statin users and nonusers similarly; therefore, they do not limit the findings on risk associations by statin use. All analyses were exploratory and should be considered as hypothesis generating. Confirmation of the results is needed from prospective randomized clinical trials.

## Conclusions

This cohort study showed that BC mortality decrease after postdiagnostic statin use was associated with a simultaneous serum cholesterol level change. This finding suggests that cholesterol-lowering interventions with statins may be beneficial for BC. Serum cholesterol should be taken into account in future studies on statin use and BC mortality.

## Notes

Supplement 1. **eMethods.** Statin Use, Cholesterol Level and Mortality Among Finnish Females With Breast Cancer: Description of Exposure and Outcome Variables **eTable 1.** Comparison Between Breast Cancer Cases Included in the Study and Those Excluded From It **eTable 2.** ICD-10 Codes Used to Detect Diagnoses From the Finnish National Registries **eTable 3.** ATC-Codes Used to Detect Drug Prescription Database of the Finnish Social Insurance Institution **eTable 4.** The Risk of Breast Cancer (BC) Death by Serum HDL and Triglycerides Levels Before and After BC Diagnosis **eTable 5.** The Risk of Breast Cancer (BC) Death by Statin Use After BC Diagnosis, Lag-Time Analysis **eTable 6.** The Risk of Overall Death by Serum Total Cholesterol and LDL Levels Before and After BC Diagnosis **eTable 7.** The Risk of Overall Death by Serum HDL and Triglycerides Levels Before and After BC Diagnosis **eTable 8.** The Risk of Overall Death by Statin Use and With Adjustment for Serum Total Cholesterol, LDL, HDL and Triglycerides Before and After BC Diagnosis **eTable 9.** Subgroup Analysis: The Risk of Breast Cancer (BC) Death by Increased Serum Cholesterol Level and Statin Use After Breast Cancer Diagnosis

Supplement 2.
Data Sharing Statement
