# Supplementary material for: Statin Use, Cholesterol Level, and Mortality Among Females With Breast Cancer
Source: JAMA Netw Open. 2023 Nov 17;6(11):e2343861. doi: 10.1001/jamanetworkopen.2023.43861 (PMC10656638; doi:10.1001/jamanetworkopen.2023.43861)
Supplement: Supplement 1. — eMethods. Statin Use, Cholesterol Level and Mortality Among Finnish Females With Breast Cancer: Description of Exposure and Outcome Variables eTable 1. Comparison Between Breast Cancer Cases Included in the Study and Those Excluded From It eTable 2. ICD-10 Codes Used to Detect Diagnoses From the Finnish National Registries eTable 3. ATC-Codes Used to Detect Drug Prescription Database of the Finnish Social Insurance Institution eTable 4. The Risk of Breast Cancer (BC) Death by Serum HDL and Triglycerides Levels Before and After BC Diagnosis eTable 5. The Risk of Breast Cancer (BC) Death by Statin Use After BC Diagnosis, Lag-Time Analysis eTable 6. The Risk of Overall Death by Serum Total Cholesterol and LDL Levels Before and After BC Diagnosis eTable 7. The Risk of Overall Death by Serum HDL and Triglycerides Levels Before and After BC Diagnosis eTable 8. The Risk of Overall Death by Statin Use and With Adjustment for Serum Total Cholesterol, LDL, HDL and Triglycerides Before and After BC Diagnosis eTable 9. Subgroup Analysis: The Risk of Breast Cancer (BC) Death by Increased Serum Cholesterol Level and Statin Use After Breast Cancer Diagnosis [file jamanetwopen-e2343861-s001.pdf]

Supplemental Online Content

Murto MO, Simolin N, Arponen O, et al. Statin use, cholesterol level, and mortality among females with breast cancer. *JAMA Netw Open*. 2023;6(11):e2343861. doi:10.1001/jamanetworkopen.2023.43861

eMethods. Statin Use, Cholesterol Level and Mortality Among Finnish Females With Breast Cancer: Description of Exposure and Outcome Variables

eTable 1. Comparison Between Breast Cancer Cases Included in the Study and Those Excluded From It

eTable 2. ICD-10 Codes Used to Detect Diagnoses From the Finnish National Registries

eTable 3. ATC-Codes Used to Detect Drug Prescription Database of the Finnish Social Insurance Institution

eTable 4. The Risk of Breast Cancer (BC) Death by Serum HDL and Triglycerides Levels Before and After BC Diagnosis

eTable 5. The Risk of Breast Cancer (BC) Death by Statin Use After BC Diagnosis, Lag-Time Analysis

eTable 6. The Risk of Overall Death by Serum Total Cholesterol and LDL Levels Before and After BC Diagnosis

eTable 7. The Risk of Overall Death by Serum HDL and Triglycerides Levels Before and After BC Diagnosis

eTable 8. The Risk of Overall Death by Statin Use and With Adjustment for Serum Total Cholesterol, LDL, HDL and Triglycerides Before and After BC Diagnosis

eTable 9. Subgroup Analysis: The Risk of Breast Cancer (BC) Death by Increased Serum Cholesterol Level and Statin Use After Breast Cancer Diagnosis

This supplemental material has been provided by the authors to give readers additional information about their work.

© 2023 Murto MO et al. *JAMA Network Open*

eMethods. Statin use, cholesterol level and mortality among Finnish females with breast cancer: Description of exposure and outcome variables

Outcome variables

Breast-cancer death

The outcome variable was determined based on causes of death registry data of Statistics Finland. Death was considered to be caused by BC if the any breast cancer ICD-10 code (C50.1-C50.9) was listed as the primary cause of death. The variable was analyzed as dichotomous; breast cancer death yes/no

Death due to any cause

The outcome variable was determined based on causes of death registry data of Statistics Finland. All recorded deaths regardless of the cause were included. The variable was analyzed as dichotomous; death yes/no

Exposure variables

Information on cholesterol and hormone receptor status

We gathered serum cholesterol, lipoprotein and triglyceride measurements and hormone receptor data from laboratory databases of Pirkanmaa, Southwest Finland and Helsinki-Uusimaa Hospital Districts

Estrogen receptor (ER), progresterone receptor (PR) and ERBB2 status in tumor tissue was extracted from pathological reports of the primary tumor and metastases. We deemed ER and PR statuses negative if < 10% and positive if ≥ 10% of tumor cells were stained; we determined the ERBB2 status negative or positive according to the pathological reports. ER, PR and ERBB2 status were included in the analysis as dichotomous variables.

All measurements for serum total cholesterol, HDL, LDL and triglycerides were obtained from the laboratory databases. We determined median cholesterol concentrations for each calendar year separately using all measurements during that year. We used the latest available median for years with no measurements. We categorized cholesterol and other lipid parameter levels as normal or elevated according to the cardiovascular recommended target levels; 193.05 mg/dL for total cholesterol,46.33 mg/dL for HDL, 115.83 mg/dL for LDL, and 150.44 mg/dL for triglyceride. To convert total cholesterol, HDL, and LDL to millimoles per liter, multiply by 0.0259; triglycerides to millimoles per liter, multiply by 0.0113.

We estimated the changes in blood cholesterol levels after the start of statin use by calculating the median cholesterol levels before and after the first year of statin purchase. Measurements from the first year of statin use were excluded as the exact time for the initiation of drug use is not known based on purchase data. In total, 980 patients began statin use after BC diagnosis. Median cholesterol level decreased subsequently in 781 participants while the level remained similar or increased compared to pre-statin purchase levels in 199 participants.

© 2023 Murto MO et al. *JAMA Network Open*

Comorbidities

Information on diagnoses of hypertension, hypercholesterolemia, coronary artery disease and diabetes mellitus (DM) during 1995-2013 were obtained from the Finnish Care Register for Health Care (HILMO). Each comorbidity was recognized based on the condition-specific ICD-10 code. The participant was categorized to have the condition (e.g. diabetes), if she had the diagnosis recorded at least once at any time during the study period. Each variable was included in the analysis as dichotomous variable.

We calculated Charlson Comorbidity Index (CCI) based on diagnoses in the HILMO database (e.g., diabetes) and drug purchase data from the Finnish Social Insurance Institution (SII) database (e.g., antidiabetic drugs). CCI was included in the analysis as continuous variable.

Information on medication use

We linked the study cohort to the national SII prescription database to collect person-level information on cholesterol-lowering, antihypertensive, antidiabetic, and anticoagulant medication purchases during 1995-2015. Usage status for each drug group was determined separately for each follow-up year based on recorded purchases. All participants remained non-users until the first year of recorded purchase. After that they were categorized as users until the end of follow-up. Post-diagnostic usage status was included into analysis as dichotomous, time-dependent variable that was updated for each follow-up year separately, starting from the year of breast cancer diagnosis. Pre-diagnosis usage was analyzed as dichotomous time-independent variable based on medication usage between 1995 and year of breast cancer diagnosis.

For statins, we also considered statin dose. We added together all statin purchases on a given year for yearly total milligram (mg) amount of use for each statin and standardized the cumulative use between different statins by dividing yearly purchased amount (mg) with drug-specific Defined Daily Dose (DDD) as listed by the World Health Organization (WHO) To estimate the yearly dosage of statin use, we calculated an intensity variable by dividing the cumulative yearly DDD amount with the cumulative duration of use. Intensity of statin use was determined separately for each follow-up year after breast cancer diagnosis, and analyzed as categorical variable stratified by tertile cut-points.

For pre-diagnosis usage, intensity of statin use was calculated by dividing statin DDDs between 1995 and year of diagnosis with number of years with recorded purchases in that same time frame. Pre-diagnosis intensity was analyzed as time-independent categorical variable stratified by tertile cut-points.

© 2023 Murto MO et al. *JAMA Network Open*

Mammography screening

We used the National Mass Inspection Registry to obtain the number of mammography screens the participants had attended before BC diagnosis. This variable was included in the analysis as continuous variable.

© 2023 Murto MO et al. *JAMA Network Open*

eTable 1. Comparison between breast cancer cases included in the study and those excluded from it.

	Breast cancer cases included in the study cohort	Breast cancer cases excluded from the study cohort
N of women	13,378	59,792
Median age at diagnosis (IQR)	62 (52-73)	62 (52-73)
Charlson comorbidity index median (IQR)	0 (0-1)	0 (0-1)
BC deaths (%)	935 (7.0%)	10,900 (18.2%)
Overall deaths (%)	2,194 (16.4%)	22,520 (37.7%)
Tumor extent at diagnosis:		
Localized (%)	7,272 (54.4%)	29,364 (49.1%)
Locally advanced (%)	4,715 (35.2%)	19,328 (32.3%)
Metastatic (%)	787 (5.9%)	5,338 (8.9%)
Unknown (%)	604 (4.5%)	5,762 (9.6%)
Tumor histology:		
Invasive ductal (%)	9,868 (73.8%)	45,180 (75.6%)
Invasive lobular (%)	2,563 (19.2%)	9,683 (16.2%)
Other (%)	945 (7.1%)	4,869 (8.1%)
Unknown (%)	1 (0,00074%)	61 (0.001%)
Primary treatment method:		
Curative-treatment surgery *(%)*	10,264 (76.7%)	38,567 (64.5%)
Other/unknown *(%)*	3,114 (23.3%)	21,225 (35.5%)

© 2023 Murto MO et al. *JAMA Network Open*

Supplementary figure 1. Flowchart for study cohort formation

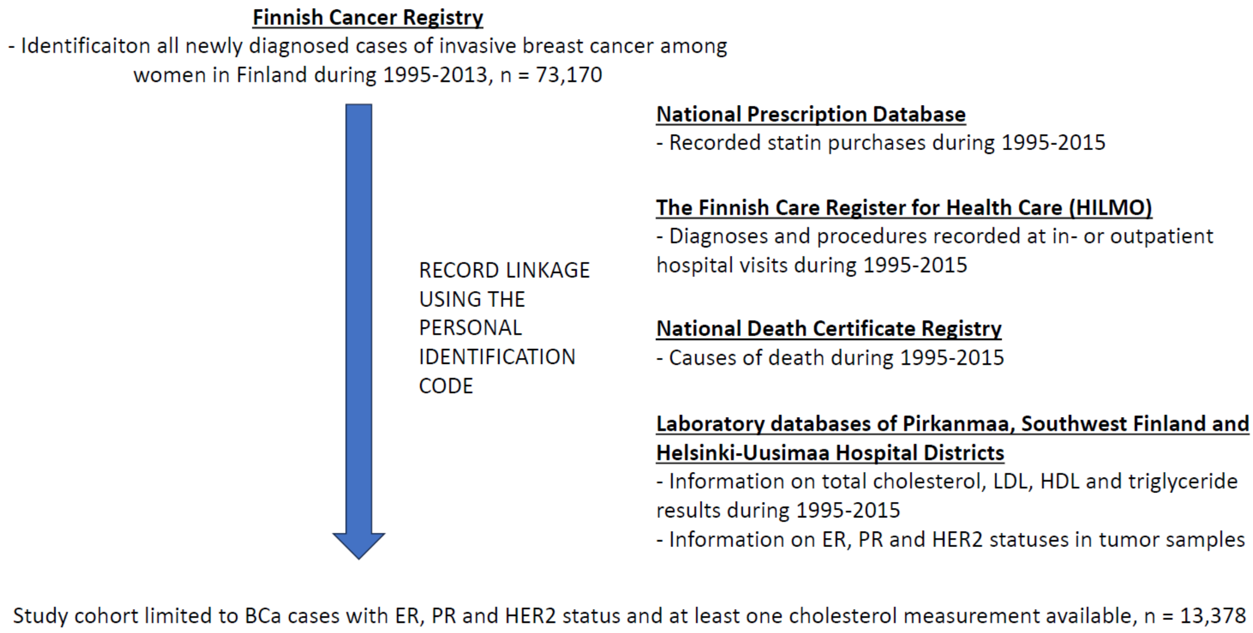

Supplementary figure 2. Categorization of statin purchases and cholesterol measurements by their temporal relation to breast cancer diagnosis

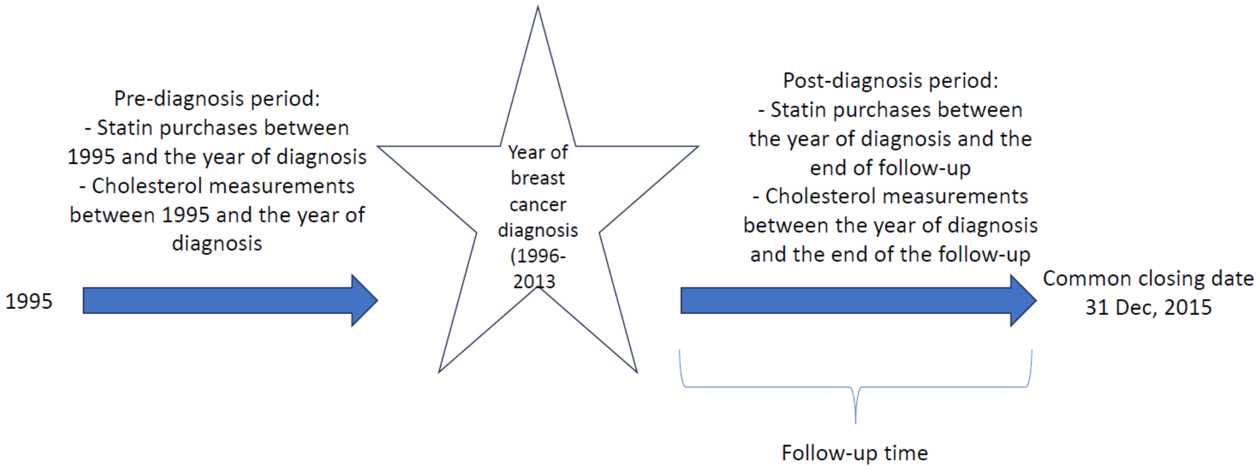

© 2023 Murto MO et al. *JAMA Network Open*

eTable 2. ICD-10 codes used to detect diagnoses from the Finnish national registries. Study cohort of 13,378 Finnish women with breast cancer (BC).

**Breast cancer**	C50.1, C50.2, C50.3, C50.4, C50.5, C50.6, C50.8, C50.9
**Diabetes**	E10, E10.00, E10.01, E10.09, E10.1, E10.2, E10.2+, E10.3, E10.3+, E10.4, E10.5, E10.6, E10.7, E10.8, E10.9<br>E11, E11.00, E10.01, E11.09, E11.1, E11.2, E11.3, E11.4, E11.5, E11.6, E11.7, E11.8, E11.9<br>E12, E12.0, E12.1, E12.2, E12.3, E12.4, E12.5, E12.6, E12.7, E12.8, E12.9, E13, E13.0, E13.1, E13.2, E13.3, E13.4, E13.5, E13.6, E13.7, E13.8, E13.9<br>E14, E14.0, E14.1, E14.2, E14.3, E14.4, E14.5, E14.6, E14.7, E14.8, E14.9<br>A2500A, 2500B, 2506A, 2506X, 2507B, 2507X, 2505A, 2506C
**Hypertension**	I10, I11, I11.0, I11.9, I12, I12.0, I12.9, I13.0, I13.1, I13.2, I13.9, I15.0, I15.1, I15.2, I15.8, I15.9
**Hypercholesterolemia**	E78, E78.0, E78.00, E78.01, E78.10, E78.11, E78.21, E78.29, E78.4, E78.40, E78.49, E78.5, E78.9, E78.1, E78.2, E78.8
**Obesity**	E65, E66, E66.0, E66.00, E66.01, E66.1, E66.2, E66.8, E66.9
**Coronary artery disease**	I25.0-I25.9

© 2023 Murto MO et al. *JAMA Network Open*

eTable 3. ATC-codes used to detect drug prescription database of the Finnish Social Insurance Institution. Study cohort of 13,378 Finnish women with breast cancer (BC).

**Antidiabetic drugs**	Metformin	A10BA02, A10BD02, A10BD03, A10BD05, A10BD07, A10BD08, A10BD10, A10BD11, A10BD13, A10BD15, A10BD20
	Phenformin	A10BA01, A10BD01
	Buformin	A10BA03
	Glibenclamide	A10BB01
	Chlorpropamide	A10BB02
	Tolbutamide	A10BB03
	glibomunidi	A10BB04
	Tolazamide	A10BB05
	Carbutamide	A10BB06
	Glipizide	A10BB07
	Gliquidone	A10BB08
	Gliclazide	A10BB09
	Metahexamide	A10BB10
	Glisoxepide	A10BB11
	Glimepiride	A10BB12, A10BD04, A10BD06
	Acetohexamide	A10BB31
	Glymidine	A10BC01
	Acarbose	A10BF01
	Miglitol	A10BF02
	Voglibose	A10BF03
	Troglitazone	A10BG01
	Rosiglitazone	A10BG02, A10BD03, A10BD04
	Pioglitazone	A10BG03, A10BD05, A10BD06, A10BD09
	Sitagliptin	A10BH01, A10BD07
	Vildagliptin	A10BH02, A10BD08
	Alogliptin	A10BD09, A10BD13, A10BH04
	Saxagliptin	A10BD10, A10BH03
	Linagliptin	A10BD11, A10BH05
	Guar gum	A10BX01
	Repaglinide	A10BX02
	Nateglinide	A10BX03
	Eksenatide	A10BX04
	Pramlintide	A10BX05
	Benfluorex	A10BX06
	Liraglutide	A10BX07
	Tolrestat	A10XA01

© 2023 Murto MO et al. *JAMA Network Open*

	Dabagliflotsil<br>Empagliflotsil<br><br><br><br>Dulaglutide<br>Eksenatide<br>Liraglutide<br>Lixisenatide<br><br>Insulins,human<br><br>Insulin, cow<br><br>Insulin, pig<br><br>Lispro<br>Aspart<br>Glul<br>Glargin<br>Determir<br>Degludek<br><br>Insulin combinations	A10BD15, A10BX09<br>A10BD20, A10BX12<br><br><br><br>A10BX14<br>A10BX04<br>A10BX04<br>A10BX10<br><br>A10AB01, A10AC01, A10AD01,<br>A10AE01, A10AF01<br>A10AB02, A10AC02, A10AD02,<br>A10AE02<br>A10AB03, A10AC03, A10AD03,<br>A10AE03<br>A10AB04, A10AC04, A10AD04<br>A10AB05, A10AD05<br>A10AB06<br>A10AE04<br>A10AE05<br>A10BX12<br><br>A10AB30, A10AC30, A10AD30, A10AE30
**Cholesterol-lowering statin drugs**	Simvastatin<br>Lovastatin<br>Pravastatin<br>Fluvastatin<br>Atorvastatin<br>Cerivastatin<br>Rosuvastatin	C10AA01<br>C10AA01<br>C10AA03<br>C10AA04<br>C10AA05<br>C10AA06<br>C10AA07
**Antihypertensive drugs**		C09AA02, C09BA02, C09BB02, C09AA16, C09AA01, C09AB01,<br>C09AA06, C09BA06, C09AA03, C09BA03, C09AA04, C09BA04, C09BB04,<br>C09AA05, C09BA05, C09BB05, C09AA10, C09BB10, C09AA08, C09BA08, C09AA07, C09BA07, C09AA09, C09BA09, C09AA11,<br>C09AA12, C09BA12, C09BB12, C09AA13, C09BA13, C09AA14, C09AA15, C09BA15,<br>C09CA02, C09DA02, C09CA06, C09DA06, C09CA01, C09DA01,<br>C09CA04, C09DA04, C09CA05, C02AC01, C02LC01, C02LC51,<br>C09CA08, C09DA08, C09DB02, C09CA07, C09DA07, C09CA03, C09DA03, C09DB01, C09DX01,<br>C02AC05, C02LC05, C02AA01, C02AA02, C02AA03, C02AA04, C02AA05, C02AA06, C02AA07,<br>C02AA52, C02AA53, C02AA57, C02AC02, C02AC04, C02AC06, C02BA01, C02BB01, C02CA01, C02LE01, C02CA02, C02CA03, C02CA04, C02CA06, C02CC01, C02CC02, C02CC03, C02CC04, C02CC05, C02CC06, C02CC07,<br>C02LF01,C02KA01, C02KB01, C02KC01, C02KD01, C09DB01, C07AB04, C07BB04, C07AB03, C07BB03, C07CB03, C07CB53, C07DB01, C07FB03,<br>C07AB05, C07AB07, C07BB07, C07AG02, C07AG01, C07BG01, C07CG01,<br>C07AB02, C07AB52, C07BB02, C07BB52, C07CB02, C07FB02,

© 2023 Murto MO et al. *JAMA Network Open*

		C07AA03, C07CA03, C07AA05, C07BA05, C07FA05, C07AB08, C07AA06, C07BA06, C07DA06, C07AB12, C07AA01, C07AA12, C07AA14, C07AA15, C07AA16, C07AA17, C07AA19, C07AA23, C07AA27, C07AB01, C07AB06, C07AB09, C07AB10, C07AB11, C07AB13, C07BA12, C07BA68, C07BB06, C07CA17, C07CA23, C03DB01, C03EA01, C03CA01, C03CB01, C03EB01, C03AA03, C03AB03, C03AX01, C02LB01, C02LC01, C02LC05, C02LC51, C02LE01, C07BA02, C07BA05, C07BA06, C07BA07, C07BA12, C07BA68, C07BB02, C07BB03, C07BB04, C07BB06, C07BB07, C07BB52, C07BG01, C07DA06, C07DB01, C08GA01, C09BA01, C09BA02, C09BA03, C09BA05, C09BA06, C09BA07, C09BA08, C09BA09, C09BA12, C09BA13, C09BA15, C09DA01, C09DA02, C09DA03, C09DA04, C09DA06, C09DA07, C09DA08, C03EA01, C03BA11, C09BA04, C03DA01, C03DB02, C03EA02, C03EA03, C03EA04, C03EA05, C03EA06, C03EA07, C03EA12, C03EA13, C03EA14, C03EB01, C03EB02, C03AA06, C03AB06, C03EA02, C03AA01, C03AA02, C03AA04, C03AA05, C03AA07, C03AA08, C03AA09, C03AA13, C03AB01, C03AB02, C03AB04, C03AB05, C03AB07, C03AB08, C03AH01, C03AH02, C03BA02, C03BA03, C03BA04, C03BA07, C03BA09, C03BA10, C03BA12, C03BA13, C03BA82, C03BB02, C03BB03, C03BB04, C03BB07, C03BC01, C03BD01, C03BX03, C03CA03, C03CA04, C03CC01, C03CC02, C03CD01, C03CX01, C03DA02, C03DA03, C03DA04, C03EA03, C03EA04, C03EA05, C03EA06, C03EA07, C03EA13, C03EA14, C03XA01, C03XA02, C08CA01, C09DB01, C09DB02, C09DX01, C09BB04, C08DB01, C08CA02, C09BB05, C07FB02, C08CA03, C08CA13, C09BB02, C08CA05, C08CA55, C08GA01, C07FB03, C08CA10, C08CA06, C08CA07, C08DA01, C08DA51, C09BB10, C08CA04, C08CA08, C08CA09, C08CA11, C08CA12, C08CA14, C08CA15, C08CX01, C08DA02, C08EA01, C08EA02, C08EX01, C08EX02, C09BB12, C02DA01, C02DB01, C02DB02, C02DB03, C02DB04, C02DD01, C02DG01,
**Hormonal antagonists**	Tamoxifen<br>Toremifen<br>Fulvestrant<br>Aminoglutetimid<br>Formestan<br>Anastrozole<br>Letrozole<br>Vorozole<br>Exemestane	L02BA01<br>L02BA02<br>L02BA03<br>L02BG01<br>L02BG02<br>L02BG03<br>L02BG04<br>L02BG05<br>L02BG06

© 2023 Murto MO et al. *JAMA Network Open*

**Anticoagulant drugs**	Warfarin<br>Fenindion<br><br>Dalteparin<br>Enoxaparin<br>Tinzaparin<br><br>Clopidogrel<br><br>Dipyridamole<br><br>Iloprost<br>Ticlopidine<br><br>Dabigatran<br>Ximelagatran<br><br>Rivaroxaban<br>Apixaban<br><br>Fondaparinux	B01AA03<br>B01AA02<br><br>B01AB04<br>B01AB05<br>B01AB10<br><br>B01AC04<br>B01AC07<br>B01AC30<br><br>B01AC11<br>B01AC05<br><br>B01AE07<br>B01AE05<br><br>B01AF01<br>B01AF02<br><br>B01AX05

© 2023 Murto MO et al. *JAMA Network Open*

eTable 4. The risk of breast cancer (BC) death by serum HDL and triglycerides levels before and after BC diagnosis. Study cohort of 13,378 females with breast cancer (BC) diagnosis in Finland from 1995 to2013.

Lipid profile status	HDL level				Triglyceride level			
	No. of BC deaths/No. of participants (%)	HR for BC death (95% CI)			No. of BC deaths/ No. of participants (%)	HR for BC death (95% CI)		
		Age-adjusted	Multivariable-adjusted[a]	Multivariable+ Statin use adjusted		Age-adjusted	Multivariable-adjusted[a]	Multivariable+ Statin use adjusted
**Lipid profile status before BC diagnosis**								
Under reference value[b]	59/807 (7.3)	1 [Reference]	1 [Reference]	1 [Reference]	372/6009 (6.2)	1 [Reference]	1 [Reference]	1 [Reference]
Over reference value[b]	421/6522 (6.5)	0.94 (0.71-1.23)	1.04 (0.78-1.39)	1.04 (0.78-1.39)	102/1298 (7.9)	1.11 (0.89-1.39)	0.99 (0.78-1.25)	0.96 (0.76-1.22)
1st tertile[c]	176/2458 (7.2)	1 [Reference]	1 [Reference]	1 [Reference]	129/2439 (5.3)	1 [Reference]	1 [Reference]	1 [Reference]
2nd tertile[c]	158/2434 (6.5)	0.93 (0.75-1.15)	0.99 (0.79-1.24)	1.00 (0.80-1.25)	165/2446 (6.7)	1.14 (0.90-1.44)	1.11 (0.87-1.41)	1.09 (0.86-1.39)
3rd tertile[c]	146/2438 (6.0)	0.85 (0.69-1.06)	0.92 (0.73-1.16)	0.93 (0.73-1.17)	180/2428 7.4	1.17 (0.93-1.47)	1.10 (0.87-1.40)	1.07 (0.84-1.36)
**Lipid profile status after BC diagnosis**								
Under reference value[b]	179/1762 (10.2)	1 [Reference]	1 [Reference]	1 [Reference]	722/10849 (6.7)	1 [Reference]	1 [Reference]	1 [Reference]
Over ref. value[b]	740/11510 (6.4)	0.71 (0.60-0.85)	0.88 (0.73-1.07)	0.88 (0.72-1.06)	191/2409 (7.9)	1.06 (0.89-1.25)	1.01 (0.84-1.21)	1.03 (0.86-1.23)
1st tertile[d]	376/4450 (8.4)	1 [Reference]	1 [Reference]	1 [Reference]	259/474 (5.8)	1 [Reference]	1 [Reference]	1 [Reference]
2nd tertile[d]	279/4418 (6.3)	0.83 (0.71-0.97)	0.92 (0.77-1.10)	0.92 (0.77-1.09)	306/4368 (7.0)	1.26 (1.06-1.50)	1.12 (0.93-1.36)	1.14 (0.94-1.37)

© 2023 Murto MO et al. *JAMA Network Open*

3rd tertile [d]	264/4408 (6.0)	0.80 (0.68-0.95)	0.98 (0.82-1.18)	0.97 (0.81-1.17)	349/4418 (7.9)	1.30 (1.10-1.53)	1.12 (0.94-1.35)	1.14 (0.95-1.38)

Abbreviations: HDL, high-density lipoprotein; HR, hazard ratio.

SI conversion factor: To convert HDL, LDL, and total cholesterol to millimoles per liter, multiply by 0.0259; triglyserides to millimoles per liter, multiply by 0.0113.

[a] Calculated using a Cox proportional hazards regression model adjusted for age at BC diagnosis, number of mammography screening rounds attended before BC diagnosis, tumor extent, tumor histological characteristics, primary treatment, coronary artery disease, diabetes, hypertension, Charlson Comorbidity Index, hormone receptor status, and use of antihormonal therapy after BC diagnosis.

[b] Cut-off values: Total cholesterol higher than 193.05; Triglycerides higher than 150.44; HDL lower than 46.33; LDL higher than 115.83 (mg/dL)

[c] HDL tertiles before BC diagnosis: first, 57.14 or less; second, higher than 57.14 to 71.62; third, higher than 71.62; Triglycerides tertiles before BC diagnosis: first, 81.42 or less;, second, higher than 81.42 to 119.47; third, higher than 119.47 (mg/dL).

[d] Total cholesterol tertiles after BC diagnosis: first, 181.47 mg/dL or less; second, higher than 181.47 to 212.36 mg/dL; third, higher than 212.36 mg/dL. Triglycerides tertiles after BC diagnosis: first, 84.07 mg/dL or less; second, higher than 84.07 to 121.68 mg/dL; third, higher than 121.68 mg/dL. HDL tertiles after BC diagnosis: first, 56.37 mg/dL or less; second, higher than 56.37 to 71.43 mg/dL; third, higher than 71.43 mg/dL. LDL tertiles after BC diagnosis: first, 100.39 mg/dL or less; second, higher than 100.39 to 128.69 mg/dL; third, higher than 128.69 mg/dL.

© 2023 Murto MO et al. *JAMA Network Open*

eTable 5. The risk of breast cancer (BC) death by statin use after BC diagnosis, lag-time analysis.

Usage of statin drugs	HR for BC death (95% CI)			
	No. of BC deaths/No. of participants (%)	Age-adjusted	Multivariable-adjusted[a]	Multivariable+serum cholesterol level adjusted
**1-Year lag-time**				
None (Reference)	565/7720 (7.3)	1 [Reference]	1 [Reference]	1 [Reference]
Any	315/5295 (5.9)	1.08 (0.93-1.25)	1.01 (0.85-1.19)	0.91 (0.77-1.08)
**3-Year lag time**				
None (Reference)	509/7450 (6.8)	1 [Reference]	1 [Reference]	1 [Reference]
Any	289/5076 (5.7)	1.05 (0.89-1.23)	1.04 (0.86-1.24)	0.92 (0.77-1.11)
**5-Year lag time**				
None (Reference)	466/7083 (6.6)	1 [Reference]	1 [Reference]	1 [Reference]
Any	272/4849 (5.6)	1.09 (0.91-1.31)	1.14 (0.93-1.38)	1.03 (0.84-1.26)

Abbreviations: HR, hazard ratio.

[a] Calculated using a Cox proportional hazards regression model adjusted for age at BC diagnosis, number of mammography screening rounds attended before BC diagnosis, tumor extent, tumor histological characteristics, primary treatment, coronary artery disease, diabetes, hypertension, Charlson Comorbidity Index, hormone receptor status, and use of antihormonal therapy after BC diagnosis.

© 2023 Murto MO et al. *JAMA Network Open*

eTable 6. The risk of overall death by serum total cholesterol and LDL levels before and after BC diagnosis. Study cohort of 13,378 females with breast cancer (BC) diagnosis in Finland from 1995 to2013.

Lipid profile status	Total cholesterol level				LDL level			
	No. of deaths/No. of participants (%)	HR for overall death (95% CI)			No. of deaths/No. of participants (%)	HR for overall death (95% CI)		
		Age-adjusted	Multivariable-adjusted[a]	Multivariable+ Statin use adjusted		Age-adjusted	Multivariable-adjusted[a]	Multivariable+ Statin use adjusted
**Lipid profile status before BC diagnosis**								
Under reference value[b]	593/3199 (18.5)	1 [Reference]	1 [Reference]	1 [Reference]	599/3279 (18.3)	1 [Reference]	1 [Reference]	1 [Reference]
Over reference Value[b]	630/4170 (15.1)	0.77 (0.69-0.86)	0.80 (0.71-0.90)	0.81 (0.72-0.91)	378/2619 (14.4)	0.87 (0.77-0.99)	0.91 (0.79-1.04)	0.91 (0.79-1.04)
1st tertile[c]	475/2485 (19.1)	1 [Reference]	1 [Reference]	1 [Reference]	409/2093 (19.5)	1 [Reference]	1 [Reference]	1 [Reference]
2nd tertile[c]	372/2448 (15.2)	0.92 (0.80-1.06)	0.89 (0.77-1.02)	0.88 (0.76-1.02)	287/1842 (15.6)	0.94 (0.80-1.09)	0.96 (0.82-1.13)	0.98 (0.84-1.15)
3rd tertile[c]	376/2436 (15.4)	0.87 (0.76-1.00)	0.84 (0.73-0.97)	0.85 (0.73-0.97)	281/1963 (14.3)	0.91 (0.78-1.06)	0.81 (0.69-0.95)	0.82 (0.70-0.97)
**Lipid profile status after BC diagnosis**								
Under reference value[b]	1267/6291 (20.1)	1 [Reference]	1 [Reference]	1 [Reference]	1118/5631 (19.9)	1 [Reference]	1 [Reference]	1 [Reference]
Over reference Value[b]	927/7087 (13.1)	0.76 (0.70-0.83)	0.88 (0.80-0.97)	0.86 (0.78-0.94)	616/4860 (12.7)	0.77 (0.70-0.86)	0.83 (0.74-0.92)	0.81 (0.73-0.91)
1st tertile[d]	1022/4648 (22.0)	1 [Reference]	1 [Reference]	1 [Reference]	818/3708 (22.1)	1 [Reference]	1 [Reference]	1 [Reference]
2nd tertile[d]	593/4351 (13.6)	0.74 (0.66-0.82)	0.83 (0.74-0.92)	0.80 (0.71-0.89)	475/3283 (14.5)	0.82 (0.73-0.93)	0.93 (0.82-1.06)	0.91 (0.80-1.03)
3rd tertile[d]	579/4378 (13.2)	0.73 (0.66-0.81)	0.88 (0.78-0.99)	0.86 (0.76-0.96)	441/3495 (12.6)	0.73 (0.65-0.83)	0.82 (0.71-0.93)	0.80 (0.70-0.92)

Abbreviations: HR, hazard ratio; LDL, low-density lipoprotein.

[a] Calculated using a Cox proportional hazards regression model adjusted for age at BC diagnosis, number of mammography screening rounds attended before BC diagnosis, tumor extent, tumor histological characteristics, primary treatment, coronary artery disease, diabetes, hypertension, Charlson Comorbidity Index, hormone receptor status, and use of antihormonal therapy after BC diagnosis.

[b] Cut-off values: Total cholesterol higher than 193.05; Triglycerides higher than 150.44; HDL lower than 46.33; LDL higher than 115.83 (mg/dL)

[c] Total cholesterol tertiles before BC diagnosis: first, 185.33 mg/dL or less; second, higher than 185.33 to 214.29 mg/dL; third, higher than 214.29 mg/dL. LDL tertiles before BC diagnosis: first, 100.39 mg/dL or less; second, higher than 100.39 to 125.87 mg/dL; third, higher than 125.87 mg/dL

© 2023 Murto MO et al. *JAMA Network Open*

[d] Total cholesterol tertiles after BC diagnosis: first, 181.47 mg/dL or less; second, higher than 181.47 to 212.36 mg/dL; third, higher than 212.36 mg/dL. Triglycerides tertiles after BC diagnosis: first, 84.07 mg/dL or less; second, higher than 84.07 to 121.68 mg/dL; third, higher than 121.68 mg/dL. HDL tertiles after BC diagnosis: first, 56.37 mg/dL or less; second, higher than 56.37 to 71.43 mg/dL; third, higher than 71.43 mg/dL. LDL tertiles after BC diagnosis: first, 100.39 mg/dL or less; second, higher than 100.39 to 128.69 mg/dL; third, higher than 128.69 mg/dL.

© 2023 Murto MO et al. *JAMA Network Open*

eTable 7. The risk of overall death by serum HDL and triglycerides levels before and after BC diagnosis. Study cohort of 13,378 females with breast cancer (BC) diagnosis in Finland from 1995 to2013.

Lipid profile status	HDL level				Triglyceride level			
	No. of deaths/No. of participants (%)	HR for overall death (95% CI)			No. of deaths/No. of participants (%)	HR for overall death (95% CI)		
		Age-adjusted	Multivariable-adjusted[a]	Multivariable+ Statin use adjusted		Age-adjusted	Multivariable-adjusted[a]	Multivariable+ Statin use adjusted
**Lipid profile status before BC diagnosis**								
Under reference Value[b]	201/807 (24.9)	1 [Reference]	1 [Reference]	1 [Reference]	918/6009 (15.3)	1 [Reference]	1 [Reference]	1 [Reference]
Over reference Value[b]	1013/6522 (15.5)	0.70 (0.61-0.82)	0.78 (0.67-0.92)	0.78 (0.66-0.92)	280/1298 (21.6)	1.15 (1.00-1.31)	1.07 (0.93-1.23)	1.06 (0.92-1.22)
1st tertile[c]	497/2458 (20.2)	1 [Reference]	1 [Reference]	1 [Reference]	288/2439 (11.8)	1 [Reference]	1 [Reference]	1 [Reference]
2nd tertile[c]	374/2434 (15.4)	1.08 (0.95-1.24)	0.88 (0.76-1.01)	0.88 (0.77-1.01)	420/2446 (17.2)	0.98 (0.85-1.14)	1.13 (0.96-1.32)	1.12 (0.96-1.30)
3rd tertile[c]	343/2438 (14.1)	1.15 (1.01-1.32)	0.85 (0.73-0.98)	0.85 (0.74-0.99)	491/2428 (20.2)	0.85 (0.73-0.98)	1.11 (0.95-1.29)	1.09 (0.94-1.28)
**Lipid profile status after BC diagnosis**								
Under reference Value[b]	502/1762 (28.5)	1 [Reference]	1 [Reference]	1 [Reference]	1702/10849 (15.7)	1 [Reference]	1 [Reference]	1 [Reference]
Over reference Value[b]	1660/11510 (14.4)	0.59 (0.53-0.66)	0.74 (0.66-0.83)	0.74 (0.66-0.83)	451/2409 (18.7)	1.05 (0.94-1.16)	0.96 (0.85-1.07)	0.97 (0.86-1.09)
1st tertile[d]	961/4450 (21.6)	1 [Reference]	1 [Reference]	1 [Reference]	633/4474 (14.1)	1 [Reference]	1 [Reference]	1 [Reference]
2nd tertile[d]	613/4418 (13.9)	0.75 (0.67-0.83)	0.86 (0.76-0.96)	0.85 (0.76-0.96)	720/4368 (16.5)	1.10 (0.99-1.23)	1.03 (0.91-1.16)	1.04 (0.93-1.17)
3rd tertile[d]	589/4408 (13.4)	0.75 (0.67-0.83)	0.95 (0.84-1.06)	0.94 (0.83-1.05)	802/4418 (18.2)	1.10 (0.99-1.23)	0.95 (0.85-1.07)	0.97 (0.86-1.09)

Abbreviations: HR, hazard ratio; HDL, high-density lipoprotein.

[a] Calculated using a Cox proportional hazards regression model adjusted for age at BC diagnosis, number of mammography screening rounds attended before BC diagnosis, tumor extent, tumor histological characteristics, primary treatment, coronary artery disease, diabetes, hypertension, Charlson Comorbidity Index, hormone receptor status, and use of antihormonal therapy after BC diagnosis.

[b] Cut-off values: Total cholesterol higher than 193.05; Triglycerides higher than 150.44; HDL lower than 46.33; LDL higher than 115.83 (mg/dL)

[c] HDL tertiles before BC diagnosis: first, ≤ 57.14, or less; second, higher than 57.14 to 71.62; third, higher than > 71.62; Triglycerides tertiles before BC diagnosis: first, ≤ 81.42 or less; second, higher than 81.42 to 119.47; third, higher than 119.47 (mg/dL)

[d] Total cholesterol tertiles after BC diagnosis: first, 181.47 mg/dL or less; second, higher than 181.47 to 212.36 mg/dL; third, higher than 212.36 mg/dL. Triglycerides tertiles after BC diagnosis: first, 84.07 mg/dL or less; second, higher than 84.07 to 121.68 mg/dL; third,

© 2023 Murto MO et al. *JAMA Network Open*

higher than 121.68 mg/dL. HDL tertiles after BC diagnosis: first, 56.37 mg/dL or less; second, higher than 56.37 to 71.43 mg/dL; third, higher than 71.43 mg/dL. LDL tertiles after BC diagnosis: first, 100.39 mg/dL or less; second, higher than 100.39 to 128.69 mg/dL; third, higher than 128.69 mg/dL.

© 2023 Murto MO et al. *JAMA Network Open*

eTable 8. The risk of overall death by statin use and with adjustment for serum total cholesterol, LDL, HDL and triglycerides before and after BC diagnosis. Study cohort of 13,378 females with breast cancer (BC) diagnosis in Finland from 1995 to2013.

Medication use status	No. of deaths/No. of participant (%)	HR for overall death (95% CI)					
		Age-adjusted	Multivariable-adjusted[a]	Additional lipid parameter adjustment			
				Total cholesterol[b]	LDL[b]	HDL[b]	Triglycerides[b]
**Usage before BC diagnosis**							
None	1573/10177 (15.5)	1 [Reference]	1 [Reference]	1 [Reference]	1 [Reference]	1 [Reference]	1 [Reference]
Any	621/3201 (19.4)	1.26 (1.14-1.39)	1.28 (1.15-1.43)	1.08 (0.96-1.20)	1.12 (1.01-1.26)	1.10 (0.99-1.23)	1.11 (0.99-1.24)
Intensity of statin use (DDD/year)							
1st tertile[c]	214/1057 (20.2)	1.12 (0.97-1.30)	1.11 (0.95-1.29)	0.97 (0.83-1.14)	1.00 (0.85-1.17)	0.97 (0.83-1.13)	0.98 (0.84-1.15)
2nd tertile[c]	212/1056 (20.1)	1.22 (1.05-1.42)	1.28 (1.10-1.50)	1.06 (0.90-1.24)	1.11 (0.95-1.31)	1.10 (0.94-1.29)	1.10 (0.94-1.29)
3rd tertile[c]	187/1057 (17.7)	1.39 (1.19-1.62)	1.46 (1.24-1.74)	1.21 (1.01-1.43)	1.27 (1.06-1.51)	1.26 (1.06-1.49)	1.26 (1.06-1.50)
**Usage after BC diagnosis**							
None	1291/7927 (16.3)	1 [Reference]	1 [Reference]	1 [Reference]	1 [Reference]	1 [Reference]	1 [Reference]
Any	903/5451 (16.6)	1.10 (1.01-1.20)	0.89 (0.80-0.98)	0.80 (0.72-0.88)	0.80 (0.73-0.89)	0.81 (0.73-0.89)	0.81 (0.73-0.89)
Intensity of statin use (DDD/year)							
1st tertile[d]	274/1628 16.8	1.03 (0.90-1.18)	0.86 (0.74-0.99)	0.78 (0.67-0.90)	0.79 (0.68-0.91)	0.79 (0.68-0.91)	0.79 (0.68-0.91)
2nd tertile[d]	259/1585 (16.3)	0.98 (0.86-1.13)	0.82 (0.70-0.94)	0.73 (0.63-0.85)	0.72 (0.62-0.84)	0.75 (0.65-0.87)	0.75 (0.65-0.87)
3rd tertile[d]	208/1603 (13.0)	1.05 (0.91-1.21)	0.82 (0.70-0.96)	0.72 (0.61-0.84)	0.72 (0.61-0.84)	0.74 (0.63-0.86)	0.74 (0.63-0.87)

Abbreviations: HR, hazard ratio; LDL, low-density lipoprotein; HDL, high-density lipoprotein; DDD, defined daily dose.

[a] Calculated using a Cox proportional hazards regression model adjusted for age at BC diagnosis, number of mammography screening rounds attended before BC diagnosis, tumor extent, tumor histological characteristics, primary treatment, coronary artery disease, diabetes, hypertension, Charlson Comorbidity Index, hormone receptor status, and use of antihormonal therapy after BC diagnosis.

[b]Cut-off values: Total cholesterol higher than 193.05 mg/dL; Triglycerides higher than150.44 mg/dL; HDL lower than 46.33 mg/dL; LDL higher than 115.83 mg/dL

[c]Statin tertiles before BC diagnosis: first, higher than0 to 111.6667 DDD/y; second, higher than 111.6667 to 192.8148 DDD/y; third, higher than 192.8148 DDD/y.

[d]Statin tertiles after BC diagnosis: first, higher than 0 to 130.6667 DDD/y; second, higher than 130.6667 to 235.2000 DDD/y; third, higher than 235.2000 DDD/y.

© 2023 Murto MO et al. *JAMA Network Open*

eTable 9. Subgroup analysis: the risk of breast cancer (BC) death by increased serum cholesterol level and statin use after breast cancer diagnosis. Study cohort of 13,378 females with breast cancer (BC) diagnosis in Finland from 1995 to2013.

Subgroup	HR for BC death (95% CI)			
	Serum cholesterol level over reference Value[a]		Statin use after BC diagnosis	
	Multivariable-adjusted[b]	Multivariable+statin use adjusted	Multivariable-adjusted[b]	Multivariable+serum cholesterol level adjusted
ER-positive (n=11179)	1.06 (0.89-1.26)	1.04 (0.87-1.24)	0.90 (0.75-1.09)	0.82 (0.68-0.99)
ER-negative (n=1641)	1.05 (0.79-1.38)	1.04 (0.79-1.37)	0.97 (0.72-1.32)	0.88 (0.65-1.19)
PR-positive (n=8896)	0.93 (0.74-1.16)	0.92 (0.73-1.14)	0.87 (0.69-1.10)	0.80 (0.63-1.01)
PR-negative (n=3830)	1.16 (0.95-1.42)	1.15 (0.94-1.41)	0.99 (0.80-1.24)	0.89 (0.71-1.11)
ERBB2-positive (n=3747)	1.07 (0.83-1.39)	1.04 (0.80-1.36)	0.85 (0.64-1.12)	0.78 (0.59-1.04)
ERBB2-negative (n=9070)	1.09 (0.91-1.31)	1.08 (0.90-1.30)	0.98 (0.81-1.20)	0.88 (0.72-1.07)
Triple-negative (n=869)	0.97 (0.67-1.39)	0.97 (0.67-1.39)	1.06 (0.72-1.57)	0.95 (0.65-1.40)
Participation in mammography screening (n=9029)	0.99 (0.79-1.23)	0.97 (0.78-1.21)	0.90 (0.71-1.14)	0.81 (0.64-1.03)
No participation in mammography screening (n=4349)	1.11 (0.91-1.36)	1.10 (0.90-1.34)	0.96 (0.77-1.19)	0.87 (0.70-1.08)
Localized BC at diagnosis (n=7272)	1.12 (0.81-1.54) p = 0.500	1.06 (0.77-1.47) p = 0.708	0.66 (0.46-0.95) p = 0.027	0.59 (0.41-0.86) p = 0.005
Locally advanced BC at diagnosis (n=4715)	1.05 (0.84-1.31) p = 0.654	1.03 (0.82-1.29) p = 0.811	0.93 (0.73-1.18) p = 0.525	0.84 (0.66-1.06) p = 0.146
Metastatic BC at diagnosis (n=787)	1.00 (0.76-1.33) p = 0.995	1.02 (0.77-1.35) p = 0.911	1.52 (1.11-2.07) p = 0.010	1.38 (1.01-1.87) p = 0.043

Abbreviations: ER, estrogen receptor; PR, progesterone receptor; HR, hazard ratio.

[a]Total cholesterol higher than 193.05 mg/dL

[b] Calculated using a Cox proportional hazards regression model adjusted for age at BC diagnosis, number of mammography screening rounds attended before BC diagnosis, tumor extent, tumor histological characteristics, primary treatment, coronary artery disease, diabetes, hypertension, Charlson Comorbidity Index, hormone receptor status, and use of antihormonal therapy after BC diagnosis.

© 2023 Murto MO et al. *JAMA Network Open*
