# Supplementary material for: Statin Use, Cholesterol Level, and Mortality Among Females With Breast Cancer
Source: JAMA Netw Open. 2023 Nov 17;6(11):e2343861. doi: 10.1001/jamanetworkopen.2023.43861 (PMC10656638; doi:10.1001/jamanetworkopen.2023.43861)
Supplement: Supplement 2. — Data Sharing Statement [file jamanetwopen-e2343861-s002.pdf]

Data Sharing Statement

Murto. Statin Use, Cholesterol Level, and Mortality Among Females With Breast Cancer. *JAMA Netw Open*. Published November 17, 2023. doi:10.1001/jamanetworkopen.2023.43861

Data
Data available: No

Additional Information
Explanation for why data not available: Patient data can not be shared due to legal regulations in Finland.
